# Case report: Combined transcutaneous spinal cord stimulation and physical therapy on recovery of neurological function after spinal cord infarction

**DOI:** 10.3389/fmed.2024.1459835

**Published:** 2024-11-06

**Authors:** Felix León, Carlos Rojas, María José Aliseda, Gerardo del Río, Eduardo Monzalvo, Adriana Pliego-Carrillo, Jimena Figueroa, Antonio Ibarra, Igor Lavrov, Carlos A. Cuellar

**Affiliations:** ^1^Centro de Investigación en Ciencias de la Salud (CICSA), FCS, Universidad Anáhuac México, Huixquilucan, Edo. de México, Mexico; ^2^Facultad de Ciencias de la Salud, Universidad Anáhuac México, Huixquilucan, Edo. de México, Mexico; ^3^Faculty of Medicine, Autonomous University of the State of Mexico, Toluca, State of Mexico, Mexico; ^4^Secretaría de la Defensa Nacional, Escuela Militar de Graduados de Sanidad, Mexico City, Mexico; ^5^Neurology Department, Mayo Clinic, Rochester, MN, United States; ^6^Kazan State Medical University, Kazan, Russia; ^7^School of Sport Sciences, Universidad Anáhuac México, Huixquilucan, Edo de México, Mexico

**Keywords:** spinal cord infarction, spinal cord injury, transcutaneous spinal cord stimulation, non-invasive neuromodulation, spinal cord stroke, paraplegia, physical therapy

## Abstract

The case of a 37-year-old woman who suffered from spinal cord infarction (SI), resulting in a complete spinal cord injury (AIS A, neurological level T10), and autonomic dysfunction is presented. This study aimed to assess the effect of transcutaneous Spinal Cord Electrical Stimulation (tSCS) on improving motor, sensory, and autonomic function after SI. During the first 8 months, tSCS was applied alone, then, physical therapy (PT) was included in the sessions (tSCS+PT), until completion of 20 months. Compared to baseline, at 20 months, an increase in ISNCSCI motor (50 vs. 57) and sensory scores (light touch, 72 vs. 82; pinprick, 71 vs. 92) were observed. Neurogenic Bladder Symptoms Score (NBSS) changed from 27 at baseline to 17 at 20 months. ISAFSCI scores in sacral autonomic function improved from 0 pts (absent function) to 1 pt. (altered function) indicating better sphincter control. EMG recordings during volitional movements, including overground stepping with 80% of body weight support showed activity in gluteus medialis, tensor fascia latae, sartorius, rectus femoris, biceps femoris, tibialis anterior, and gastrocnemius medialis, indicating a partial reversion of paralysis. RMS analysis indicated higher activity during “tSCS on” compared to “tSCS off” during overground stepping in bilateral rectus femoris (*p* < 0.001) and gastrocnemius medialis (*p* < 0.01); and unilateral biceps femoris, and tibialis anterior (*p* < 0.001). As this is the first report on the use of tSCS in the case of SI, future studies in a case series are warranted.

## 1 Introduction

Spinal Cord Stroke or Spinal Cord Infarction (SI) is a rare condition that represents only 0.3%−1% of all vascular neurological conditions (1). An acute disruption of the spinal cord blood supply may result in ischemia and infarction, triggering a wide range of neurological disorders related to the vascular territory affected (2). Cohort studies reported that 45% of SI were related to perioperative procedures (i.e., aortic aneurism surgery and endovascular repair) (3), and 1.8% related to epidural anesthesia (4). Idiopathic SI cases occur in 20%−40% of the cases. In addition, MRI abnormalities are not always found (5). The severity of symptoms at a nadir after SI is related to poor prognosis, including paralysis, bladder catheterization, and pain (3–6). Reports of SI based on MRI analysis have shown anterior spinal cord compromise (ventral horn) as the most common injury pattern, while transverse infarctions are rare. In any case, more than two spinal levels are commonly affected, including in the most severe cases, from thoracic to conus medullaris (3, 7).

Transcutaneous Spinal Cord Electrical Stimulation (tSCS) is a non-invasive neuromodulation strategy aimed at restoring motor, sensory, and autonomic functions after Spinal Cord Injury (SCI) (8). Even though studies with small samples have been published, improvements in motor function (i.e., posture, volitional movements, and reduction in spasticity) have been consistently reported among the participants (9–12). Additionally, improvements in bladder capacity, voiding, and anorectal function have also been reported (13–15). To our knowledge, no studies evaluating the effect of tSCS on functional restoration after SI have been reported to date. This study aimed to evaluate the effects of tSCS on motor, sensory, and autonomic functions in a patient with complete paraplegia (flaccid paralysis, AIS-A) due to SI. Results are presented for 20 months (8 months with tSCS alone and 12 months with tSCS+) Physical therapy (PT) with evaluations every 4 months.

## 2 Case description

**Figure 1:**
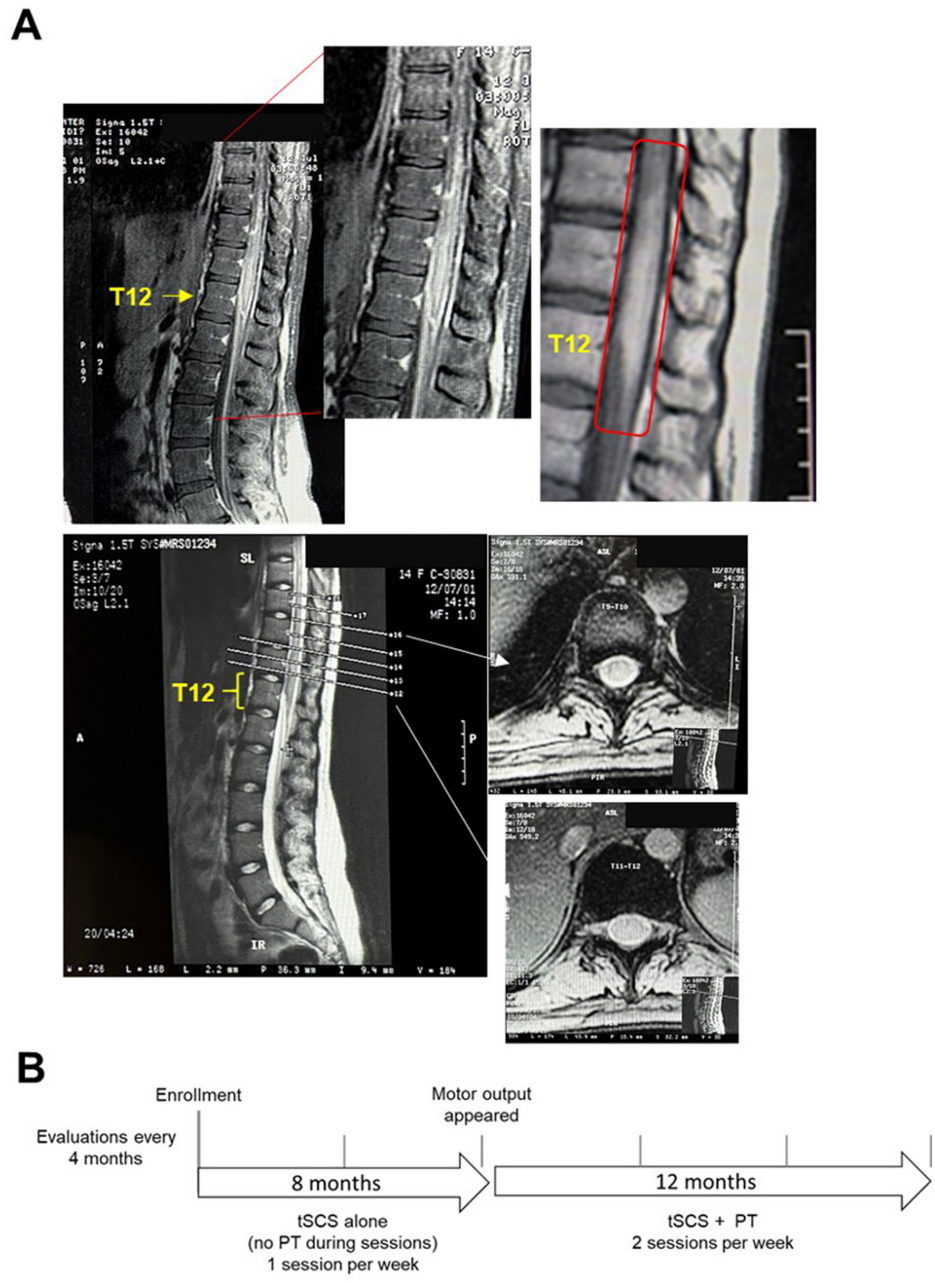
**(A)** 1.5 T MRI performed after a surgical procedure (2001) revealed an injury in T9–T12 compatible with an SI. The upper panel shows a sagittal slide and a zoomed image on the right corresponding to the lumbar enlargement. The image on the right also shows radiological abnormalities. In the lower panel, coronal slides are shown at T9–10 (upper), and T11–12 (lower) extracted from the image at the left. Findings included epiduritis and edema, which were interpreted by the radiologist. **(B)** The diagram showing timeline of the protocol. During the first 8 months, tSCS was applied alone, without PT. After motor output appeared around 7 months of tSCS alone, tSCS+PT was included for the following 12 months. Evaluations were applied at baseline and every 4 months.

On June 23^rd^, 2001, the patient was admitted to emergencies after reporting nausea, right iliac fossa pain, anorexia, and fever. After clinical screening, the participant was scheduled for an open appendectomy. Consequently, an epidural block was performed at the T11–T12 level. During the postoperative hours, the patient regained consciousness and complained of loss of sensation, and weakness in both lower limbs, and absence of uresis. After 24 h, symptoms continued to evolve until the patient finally developed paraplegia. The neurological evaluation determined a complete SCI, level T10–T11 for all sensory modalities with muscle strength 0/5. Achillean and patellar reflexes were absent. A Gadolinium-based contrasted MRI (1.5 T) was subsequently performed. The findings included dorsal-lumbar syrinx (T6–T11), multiple cord injuries and epiduritis of chemical or traumatic etiology between T3–T11, and spinal edema from T8 to the medullary cone, probably related to (as indicated by the radiological interpretation) chemical myelitis due to the presence of medication in the arachnoid space, spinal cord ischemia due to vasospasm secondary to drug-induced arachnoiditis or spinal edema secondary to trauma (Figure 1A). Days after the patient was discharged with a diagnosis of SI related to an epidural anesthesia procedure. Since the diagnosis of SI, the patient attended PT sessions (5 days per week) during the first 10 years. The PT program included muscle electrostimulation, trunk exercises and passive mobilization in lower limbs. After this period, the patient gained trunk control and improved transfers. Subsequently, PT was stopped for the following 6 years. Finally, during the last 6 years (except for 2019 and 2020 due to the COVID-19 pandemic), the patient received PT in combination to muscle electrostimulation 3 days per week. None of the previous therapies have led to significant improvements in motor, sensitivity, or autonomic function below T10.

## 3 Diagnostic assessment

The patient was enrolled on May, 2022, under the protocol “*Evaluation of Transcutaneous Spinal Cord Stimulation on the Autonomic Functions and Quality of Life in Spinal Cord Injured Subjects”* approved by the Ethics Committee of the Universidad Anáhuac México (Code 202209). The patient signed the inform consent. During the first 8 months, PT was not included during tSCS sessions due to: (a) the patient was attending PT by her own, and was decided to prevent fatigue, and (b) the primary aim was to improve autonomic and sensory function. Due to lower limb motor improvements around 8 months, tSCS+PT was implemented during sessions the following 12 months (Figure 1B).

The patient (37 years-old, 36 kg, 1.54 cm) presented severe muscle atrophy in lower limbs and reported frequent urinary infections (UI) leading to hospitalizations twice a year. The last UI requiring treatment occurred 1 month before enrollment. For bladder emptying, the patient indicated the use of clean intermittent catheterization. During the 1^st^ week, evaluation of motor and sensory function (ISNCSCI) was performed. The SCI was classified as complete [AIS-A; neurological level (NL), T10]. *Right/left* motor (Upper Extremity Motor Score, UEMS; Lower Extremities Motor Score, LEMS) and sensory scores (Light Touch, LT and Pinprick, PP) at baseline (BL) are shown in Table 1. The autonomic evaluation (ISAFSCI) resulted in a total score of 11: cardiovascular, thermoregulation core body temperature and broncho-pulmonary system were found normal (2 pts. each), while the sudomotor response was found altered (1 pt.), resulting in hypohidrosis below the NL. The sacral autonomic evaluation indicated absent (0 pts.) or altered (1 pt.) functions in the bladder, bowel, and sexual function.

**Table 1:** ISNCSCI motor and sensory scores at baseline and every 4 months until 20 months completion of the intervention.

**Motor and sensory scores (ISNCSCI)**						
**Variable**	**2022**	**Months After Beginning of tSCS**				
	**BL**	**4**	**8** ^*^	**12**	**16**	**20**
**Motor**						
**UEMS**						
Right	25	25	25	25	25	25
Left	25	25	25	25	25	25
**UEMS Total**	**50**	**50**	**50**	**50**	**50**	**50**
**LEMS**						
Right	0	0	1	3	3	4
Left	0	0	1	2	2	3
**LEMS total**	**0**	**0**	**2**	**5**	**5**	**7**
**Sensory**						
**Light touch**						
Right	35	42	39	41	45	41
Left	37	43	41	42	42	41
**LT total**	**72**	**85**	**80**	**83**	**87**	**82**
**Pinprick**						
Right	36	41	39	41	44	46
Left	35	44	41	41	43	46
**PP Total**	**71**	**85**	**80**	**82**	**87**	**92**

ISNCSCI, International Standards for the Neurological Classification of the Spinal Cord Injury; tSCS, transcutaneous Spinal Cord Stimulation; BL, baseline; UEMS, Upper extremities motor scores; LEMS, Lower extremities motor scores; LT, Light touch; PP, Pinprick. ^*^PT was including in combination with tSCS.

Spinally Motor Evoked Potentials (SMEPs) were recorded bilaterally with the patient lying in supine position (10 kHz, LabChart 8, ADInstruments^®^) in the gluteus medialis (Glu), sartorius (S), vastus medialis (Vm), rectus femoris (RF), biceps femoris (BF), tibialis anterior (TA), and gastrocnemius medialis (Gm) muscles. Biphasic square pulses (500 μs per phase, 0.1 Hz, 1–28 mA, 1 mA steps, DS8R, Digitimer^®^) were applied by pairs of electrodes (2.5 cm diameter, MedStar) placed at T11–T12 and T12–L1 as cathodes, and electrodes (4 × 8 cm MedStar) placed bilaterally in the iliac crests as anodes. No SMEPs were observed at any muscle at BL. Surface electromyography (EMG) was recorded bilaterally in RF, BF, TA, and Gm during efforts at volitional movements in conjunction with the Jendrassik maneuver during “tSCS off” and “tSCS on” (2 kHz, 20–500 bandpass, LabChart, ADInstruments^®^). Electrode positioning for EMG recordings are shown in Supplementary material 3. To reduce stimulus-induced artifacts, the following process was carried out: first, two Notch filters were applied, one with a central frequency of 30 Hz and the other at 60 Hz, both with a 6 Hz bandwidth. Then, a bandpass filter was used, with a bandwidth ranging from 10 Hz to 500 Hz. A peak detector with a 60-sample lag was applied to the resulting signal to detect any residual artifact at the stimulation frequency. Once the 30 Hz peaks were identified, 7 points before and 7 points after each peak were removed. Subsequently, the 14 points (7 ms) were replaced with zeros. The signal was then processed using a moving average filter with a 10-point window, and the envelope was extracted. This method was inspired by the approach described in Hofstoetter et al. (16). Finally, the RMS (Root Mean Square) value was calculated for both “tSCS off” and “tSCS on” recordings, adjusting their lengths to match the shortest overground stepping recording, which lasted 17.45 seconds. Artifact removal, RMS and the EMG envelopes were generated in MatLab^®^ (R2023b). Spectral analysis was performed to determine the presence of stimulus artifacts and components of EMG for each muscle.

No EMG activity was detected in any muscle when the patient was instructed to perform hip extension, flexion, adduction (ADD), and abduction (ABD); knee flexion/extension and plantar flexion/extension at BL with “tSCS off” and “tSCS on.” Bilateral H-reflexes evoked at the popliteal fossa (1 ms square pulses, 0.1 Hz, bipolar stimulation, DS8R Digitimer^®^) and recorded (10 kHz, LabChart, ADInstruments^®^) at the soleus muscle were absent as well. Due to the lack of information on tSCS on SI, we decided conservatively to start with a single weekly session for 4 months, except for a 2-week period where the patient tested COVID-19 positive. The Neurogenic Bladder Symptoms Score (NBSS), ISNCSCI, ISAFSCI, and SMEPs were evaluated at baseline and every 4 months until completion of 20 months. The set-up for SMEPs (see above) was used for the tSCS protocol.

tSCS has been applied to restore motor function in traumatic SCI. Parameters include stimulation at 30 Hz delivered at T11–T12 and T12-L1. Acute and chronic effects of tSCS have been documented and include improvements in sit-to-standing (10, 17), and voluntary motor activity (18) including stepping (16, 19). Gerasimenko et al. (20) employed tSCS delivered at T11 alone or in combination with Coxigeal stimulation to facilitate rhythmic step-like movements. Subjects were also treated with buspirone in combination with tSCS (20). tSCS aimed to improve autonomic dysfunction was chosen based on previous reports. Phillips et al., applied tSCS at 30 Hz to reduce orthostatic hypotension in four individuals with autonomic dysfunction. A single cathode was placed between T7–T8 spinous process (21). In a case report, Sachdeva et al. (22), delivered tSCS at 30 Hz to ameliorate cardiovascular dysfunction after anorectal stimulation. These authors placed the cathode at the interspinous processes T7/8 (22). In a case series, Kreydin et al., recorded anorectal contractions in acute testing (3 subjects) and chronically in 1 subject during tSCS at 30 Hz (cathode in T11–T12 and L1-2) (15). The same group employed stimulation at 30 Hz (T11–T12, L1-2) during 24 sessions in 8 weeks aimed to reduce neurogenic bladder symptoms and their consequences in 5 subjects with SCI (23). According to the above, pulses at 30 Hz were applied to complete 30 min per session with the patient lying in supine position. Current intensity was selected based on the subject's tolerance and minimal contractions in the abdominal muscles. Current intensities had a range between 15–22 mA. The subject did not experience unpleasant sensations or pain during the stimulation procedure. No responses in any muscle were observed at BL.

### 3.1 Motor evaluation

**Figure 2:**
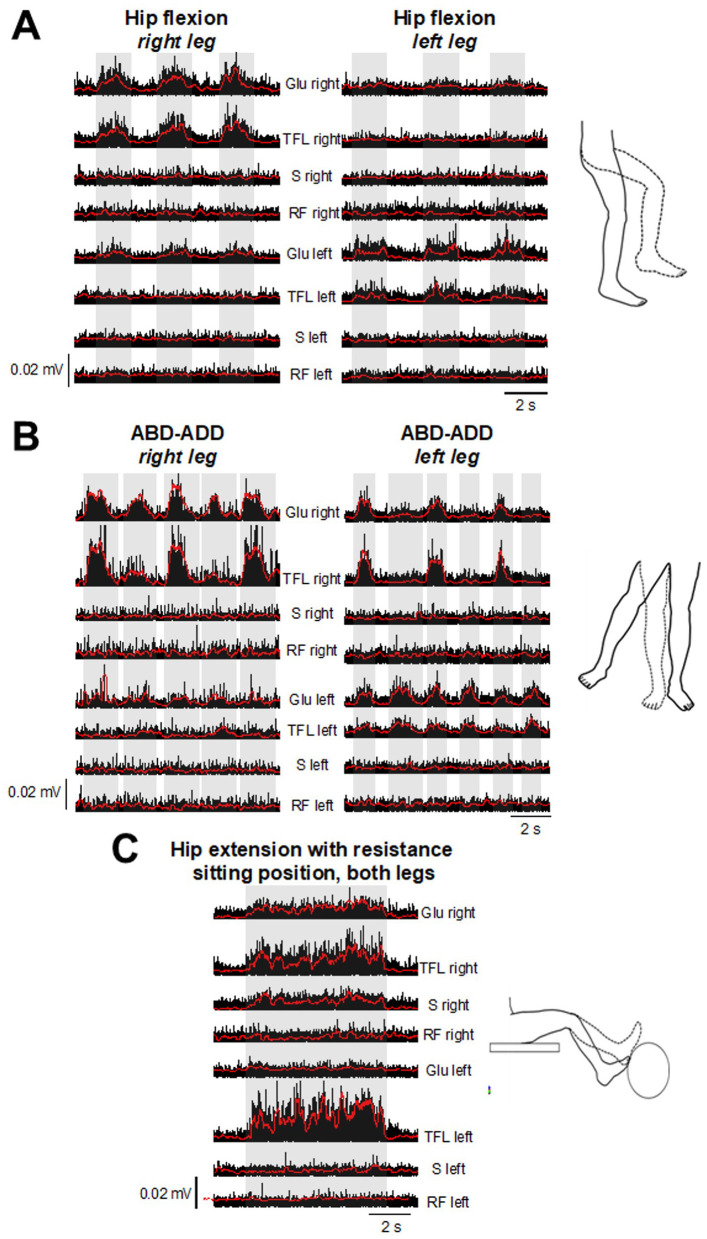
After 6 months of tSCS+PT (14 months from the beginning of the protocol), EMG testing with “tSCS off” showed bursting activity during attempts of hip flexion **(A)**, alternating abduction, and adduction **(B)** in Glu, and TFL. **(C)** Slight resistance during hip and knee extension in both legs revealed activation in S. Bilateral RF did not exhibit activity in any exercise. Corresponding EMG envelopes are also shown (red traces). Glu, gluteus medialis; TFL, tensor fascia latae; RF, rectus femoris; S, sartorius.

**Figure 3:**
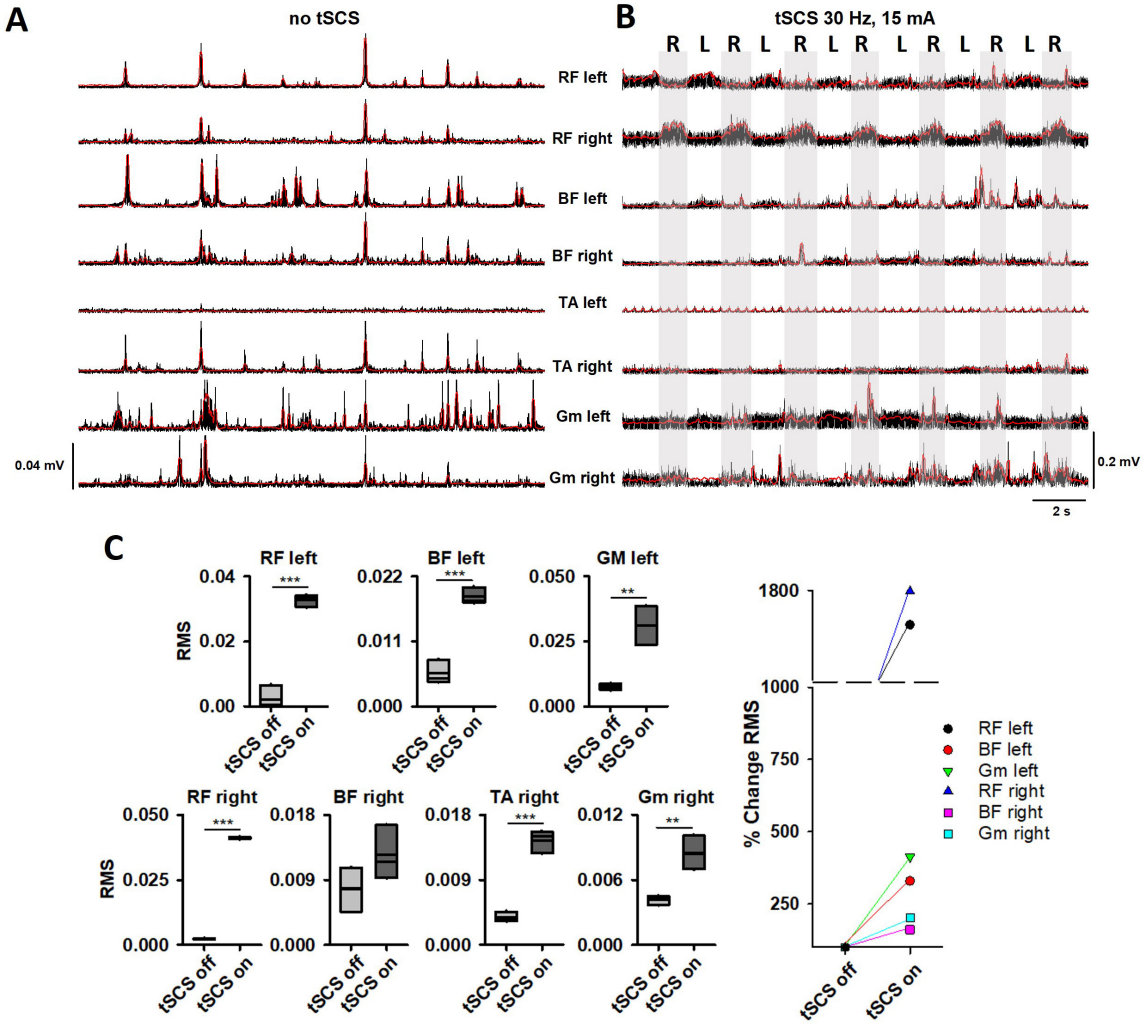
EMG and corresponding envelopes (red traces) during overground stepping with 80% of BWS are shown in bilateral RF, BF, TA, and Gm, with “tSCS off” (no tSCS) **(A)** and “tSCS on” (30 Hz, 15 mA) **(B)** at 10 months of tSCS+PT (18 months from the beginning of the protocol). **(C)** Comparisons of RMS with “tSCS off” and “tSCS on” from recordings in **(A, B)**. Statistically significant difference was found in all tested muscles (**p < 0.01, ***p < 0.001). The percentage of change (%) of the RMS is shown in the right plot. RF, rectus femoris; BF, biceps femoris; TA, tibialis anterior; Gm, gastrocnemius medialis.

No motor responses were found in both legs during the first 7 months of the protocol (0 pts. *LEMS Total*). Early during the 8^th^ month, when tSCS was still applied without PT, mild hip contractions were noticed. Consequently, LEMS changed from 0 pts. to 2 pts. at 8 months (Table 1). Based on motor improvement, tSCS+PT was implemented during sessions twice a week (Figure 1B). The goal of PT was to train lower limb extension movements (hip and knee extension) and trunk control to enable upright standing and overground stepping with 80% of body weight support (BWS) (Super Grúa, El Abuelo Cómodo^®^). The progression of PT exercises was divided into phases according to improvements in the subject (Supplementary material 1). Due to sustained motor improvements in hip muscles during the following months (Table 1), postural control and standing and overground stepping with 80% of BWS exercises were included (Supplementary material 1). Motor scores increased 5 pts. *(LEMS Total)* after 12 months (8 months of tSCS alone and 4 months with tSCS+PT), maintaining the same scores in the next evaluation (8 months tSCS alone and 8 months with tSCS+PT) (Table 1). Motor improvements around this time (12–16 months) led to the participant to perform ABD, ADD, and flexion of the hip during standing with BWS even with “tSCS off.” Rectified EMGs with “tSCS off” and corresponding envelopes in bilateral Glu, RF, S and tensor fasciae latae (TFL) are shown in Figure 2 (Electrode positioning is shown in Supplementary material 3). For each movement, the subject was instructed to perform 5 attempts in each leg, highlighted by the gray bars in Figures 2A, B. Glu and TFL exhibited bursting activity during hip flexion attempts in both legs, while S, and RF did not show activity (Figure 2A). During ABD-ADD attempts in the right leg, *right* Glu and *right* TFL showed bursting activity, as well as *left* Glu and *left* TFL although with lower amplitude. ABD-ADD attempts on the left leg also showed EMG bursting activity in *left* Glu, and *left* TFL, as well as *right* Glu and *right* TFL. S and RF on both legs were silent during ABD-ADD attempts (Figure 2B). In the same assessment, a movement consisting of pushing a resistance with both legs (exercise ball) during sitting position also showed EMG bursting activity in bilateral Glu and TFL and also revealed activity in the left S (Figure 2C, gray bar). Next, overground stepping with 80% of BWS was implemented during tSCS+PT sessions. A physiotherapist propelled the BWS system for safety reasons, but no assistance was provided for leg movement (Supplementary Video). EMG was recorded bilaterally in RF, BF, TA, and Gm at 10 months with tSCS+PT and 8 months with tSCS alone. With “tSCS off,” EMG activity appeared in bilateral RF, BF, Gm, and *right* TA, although no alternation was observed between the legs (Figure 3A). In addition, TA left did not show activity during “tSCS off.” After stimulus artifact removal, during “tSCS on,” an alternating pattern emerged bilaterally in RF, but not in the rest of the recorded muscles (Figure 3B). Gray bars in Figure 3B highlight stepping-like attempts on the right leg (R). As a periodic component persisted after artifact removal in TA left (Supplementary material 4), this muscle was excluded from RMS analysis. Comparison of the RMS with “tSCS on” vs. “tSCS off” during overground stepping showed statistical differences in RF left (*p* < 0.001), BF left (*p* < 0.001), GM left (*p* < 0.01), RF right (*p* < 0.001), TA right (*p* < 0.001), and Gm right (*p* < 0.01). No statistical differences were found in BF right (*p* > 0.05) (Figure 3C). TA left that was excluded from analysis due to persistent periodic artifact and absence of EMG spectral components (Supplementary material 4). The right panel in Figure 3C compares the percentage of change in RMS and demonstrate higher values during “tSCS on” vs. “tSCS off” in *right* and *left* muscles. In *left* RF and *right* RF, the difference was more than 1,000%, while in *right* Gm and *left* Gm were 202% and 412%, respectively. In the *right* TA, and *left* BF, the difference in the RMS was 384% and 329%, respectively. Finally, in *right* BF the difference was 161% (Figure 3C, right panel). Finally, motor scores at 20 months (8 months with tSCS alone and 12 months with tSCS+PT) increased in the *right leg* (4), and *left leg* (3) as shown in Table 1. ISNCSCI motor assessments from BL to 20 months follow-up are shown as Supplementary material 2. Single pulse stimulation delivered at Th12-12, Th12-L1 did not show SMEPS during the intervention. Bilateral H-reflexes recorded at soleus muscles were absent at all time points.

### 3.2 Sensory evaluation

Improvements in sensory function were observed after 4 months of tSCS alone compared to BL, in *LT Total* (72 pts. vs. 85 pts.), and *PP Total* (71 pts. vs. 85 pts.). At 8 months, *LT* sensory scores slightly decreased compared to 4 months: *LT Total* (80 pts.), *PP Total* (80 pts.). In the following evaluations (with tSCS+PT), sensory scores continuously improved at the 12^th^ month (*LT Total*, 83 pts., *PP Total*, 82 pts.), and 16^th^ month (*LT Total*, 87 pts., *PP Total*, 87 pts). The final evaluation in the 20^th^ month revealed a decrease in light touch compared to the previous evaluation (*LT Total*, 82 pts.), but an increase in *PP Total* (92 pts.) (Table 1). ISNCSCI sensory assessments from BL to 20 months follow-up are shown as Supplementary material 2.

### 3.3 Autonomic function evaluation

NBSS total score decreased from 27 (BL) to 17 (20 months), representing a significant clinical change (24). In general, the patient reported an improvement of sphincter control, and continence, eliminating the recurrence of UI. Sacral Autonomic Function (ISAFSCI) changes from BL to 20 months included: improved ability to prevent bladder leakage (0 vs. 1), awareness of bowel fullness (0 vs. 1), and ability to prevent bowel leakage (0 vs. 1).

## 4 Discussion

The cause of SI can be defined in < 50% of the cases of this condition (1). Partial or total recovery occurs after a few months, depending on the severity of the injury (3–6). In this case report, we present a patient who suffered an SI 23 years ago. Even though trunk control improved after many years with PT, the patient's diagnosis was discouraging as minimal improvements in motor, sensory, or autonomic functions below T11 following the SI occurred. Due to a lack of reports on tSCS in SI, we implemented a conservative, exploratory protocol consisting of one session per week, considering that the participant was attending PT on her own (3 times per week), absence of SMEPs and H reflex, flaccid paralysis, and multiple radiological findings from T3 to conus medullaris (see “Case Report”). To the best of our knowledge, this is the first case of an SI in an AIS-ASIA grade A subject treated with tSCS.

Motor, sensory, and autonomic improvements after traumatic SCI have been reported with tSCS (10–19, 23). tSCS has demonstrated improvement in urinary function with 3 sessions per week during 8 weeks in patients that suffered traumatic SCI (23), and in anorectal function in three subjects and one participant sub-chronically (5 days per week, during 5 weeks) (15). Other studies, including AIS-A and B patients, also demonstrated motor improvement in upper and lower limbs during tSCS applied between 1 to 4 months (12). Although the participant in this case report received fewer sessions per week compared to other studies, our results suggest that even a low number of sessions per week could have a positive impact on a long-term following. After 4 months of tSCS alone, the patient noticed improvements in sensory function, specifically in the hip (L1 dermatome), pelvic floor, and gluteal region (L1, S3). Improvements were more evident after 8 months of tSCS alone and coincided with moderate motor output improvements in hip muscles (Table 1). Consequently, a tSCS+PT program was started, and improvements led to EMG bursting during motor testing, even in the absence of tSCS (Figure 2). Interestingly, muscle activity shown in Figure 2 does not correspond to typical muscle patterns. For example, during right hip flexion (Figure 2A), extensor muscle Glu and weak hip abductor TFL were active, as well as the Glu left. The same pattern was observed during hip flexion in the left leg (Figure 2A), where left Glu, TFL became active, as well as right Glu. Right Glu and TFL were active during ABD-ADD attempts in the right leg. Notably, during ABD-ADD attempts in the left leg, right Glu, TFL were active along with left Glu, TFL (Figure 2B). During movement execution as shown in Figure 2C, right S became active, including right Glu and TFL and left Glu. Although several muscles were tested during the hip abduction/adduction/flexion and extension movements, the muscles shown in Figure 2 were the only muscles active around the 16^th^ month. After overground stepping with 80% of BWS was included as part of tSCS+PT sessions, bilateral RF, BF, and right TA and Gm exhibited activity, although the emerging pattern did not correspond to a typical stepping pattern (Figure 3, Supplementary Video). For example, during steps in the right leg, antagonist RF and BF and TA and Gm were coactive. In the left leg, RF and BF were coactive as well while no activity was observed in the left TA and Gm (Figure 3). During movement attempts, both shown in Figures 2, 3, the subject had difficulties to command the muscles, and considering that some muscles became active after 22 years of paralysis, it is conceivable that a typical motor pattern can be altered not only at spinal cord level but in supraspinal structures. The evaluation of motor pattern execution including supraspinal commands is far beyond the scope of this case report, and will be analyzed in the future. In addition, sustained sensory improvements (light touch and pinprick) were also observed (Table 1).

tSCS aims to activate spinal circuits below the injury level, providing augmentation of the excitability to the cervical (11, 25, 26), lumbar enlargements (16, 17, 27), or both (19). It is possible that changes in autonomic and sensory function during the first 8 months with tSCS alone in addition to PT (3 times per week on her own) contributed to motor improvements observed later. In previous studies, tSCS was applied in cervical or lumbar enlargements, allowing that preserved, but “dormant” circuits can be activated through tSCS (8, 11, 20, 25). In our study, tSCS was applied at T11 to L1, corresponding to the levels affected by the SI (see “Case Report” and Figure 1A). Motor, sensory, and autonomic improvements in the patient may be explained by residual networks that subserved the mechanisms of neural restoration. Animal and human studies with electrical spinal stimulation have described potential mechanisms for spinal cord restoration, including plasticity, re-engaging neurons that lost supraspinal input, generation of new connections [reviewed in (28, 29), and recently, a reduction of excitability driven by a specific subpopulation of neurons that promote stepping recovery have been shown (30)]. These mechanisms seem to be facilitated by locomotor training, engaging residual supraspinal inputs to the spinal cord (31).

In this case report, no H-reflex (and myotatic reflexes below injury level) or SMEPs were observed at any time point. Motor thresholds depend on spinal level stimulation at the lumbar enlargement, according to motor pool distribution (27, 32). Previous reports in healthy subjects describing spatial-temporal characteristics of SMEPs components used tSCS currents up to 100 mA; however, motor thresholds were achieved around 20 mA, stimulating T11–T12 and T12-L1 in tibialis anterior and medial gastrocnemius as shown in (27). In this case report, the maximal intensity applied for SMEPs testing was 28 mA at 0.1 Hz. Current intensities above 20 mA were perceived as unpleasant by the subject, limiting perhaps the appearance of SMEPs in recorded muscles. In this sense, one limitation of this study is that the current intensity applied during tSCS was not selected based on the threshold of motor-evoked responses as reported in previous reports. Interestingly, muscles tested during SMEPs in this study including RF, BF, and GM exhibited bursting activity after 18 months (Figure 3). Emeliannikov et al., found that in injuries at Th10-L2, SMEPs as well as H reflex were absent in 3 out of 10 subjects (33). Similarly, Shapkova et al., did not find H-reflex in 7/19 subjects with and SMEPs were absent in 2 out of 19 participants, although details on AIS, and neurological level of these 2 subjects were not provided (34). The absence of evoked potentials could be explained by several factors (not mutually exclusive): reflex responses (i.e., SMEPs) could be limited by still dormant spinal circuits, partial damage in motor pools, concomitant chronic injury in peripheral nerves and muscles and likely due to cauda equina syndrome (35, 36). Additional studies correlating SI areas (imaging) and muscles showing activity (electrophysiology) will be performed in the following months. Electrophysiological testing (SMEPs, EMG, and H-reflex) will also be carry out in the future.

Bladder and anorectal function improvements with the use of tSCS have been reported (13, 15, 23). Although in this study we did not evaluate objective parameters, the patient reported a reduction in leaks and improvements in continence both in bladder and bowel function, replicating results from those studies. Importantly, the severity and frequency of UI were reduced, particularly for the last 8 months of tSCS+PT. Burning pain in lower limbs was occasionally perceived in some areas of lower limbs. Pain did not appear during or immediately after tSCS sessions, lasted no more than 1 day, and resolved without medication. We may speculate that pain episodes may be related to the following central mechanisms: transient, aberrant connections in the dorsal horn, plastic changes related to dorsal horn neuron spontaneous firing, sensitization due to continuous afferent input (reviewed in West et al. (37)), and a peripheral mechanism related to muscle fatigue (muscle soreness).

The therapeutic mechanisms of tSCS on motor, sensory, and autonomic function after SCI remain elusive, although recent findings have been reviewed (38–40). Findings from animal studies have provided some insights into plastic changes in the nervous system, mainly derived from epidural stimulation (ES). For example, excitatory interneurons located in the intermedia laminae of the spinal cord have shown re-organizing properties relevant for walking recovery (30). ES also promoted a higher count of neural cells and subcellular markers associated to regeneration and recovery in a rodent model of SCI (41). Since the basic mechanisms of tSCS are similar to those of ES (32, 42, 43), tSCS may exhibit comparable properties in restoring lost functions after SCI. In addition, it was recently described that tSCS reduces hyperreflexia, spasticity and enhances motor output in a rodent model of SCI explained by a prevention of chloride imbalance related to cotransporters in the spinal cord (44). Although a clinical case, the results presented here show that the use of tSCS could be explored in other lower motor neuron syndromes, which present symptoms such as hyporeflexia, weakness, and muscle atrophy similar to those exhibited by the subject of this study. Although the classification of lower motor neuron syndromes includes hereditary, immune, and even sporadic patterns (45), patients suffering from these diseases could benefit from tSCS in the future.

## 5 Conclusion

This is the first report of tSCS used as a therapy to ameliorate motor, sensory, and autonomic dysfunction following SI. Improvements in motor, sensory (light touch and pinprick), and autonomic functions (bowel and bladder) were observed after 22 years of paraplegia with tSCS applied alone (the first 8 months) and with combined tSCS+PT during the following 12 months. No serious adverse events of tSCS were noticed. Bilateral EMG activity were observed, indicating a partial reversion of paralysis.

## Data Availability

The raw data supporting the conclusions of this article will be made available by the authors, without undue reservation.
