# Supplementary material for: Case report: Combined transcutaneous spinal cord stimulation and physical therapy on recovery of neurological function after spinal cord infarction
Source: Front Med (Lausanne). 2024 Nov 6;11:1459835. doi: 10.3389/fmed.2024.1459835 (PMC11576297; doi:10.3389/fmed.2024.1459835)

***Supplementary Material***

Case Report: Combined Transcutaneous Spinal Cord Stimulation and Physical Therapy on Recovery of Neurological Function after Spinal Cord Infarction

**Felix León, Carlos Rojas, María José Aliseda, Gerardo del Río, Eduardo Monzalvo, Adriana Pliego-Carrillo, Jimena Figueroa, Antonio Ibarra, Igor Lavrov and Carlos A. Cuellar***

*** Correspondence:** Carlos A. Cuellar: carlos.cuellarra@anahuac.mx

# Physical Therapy

Physical Therapy: The following is a sample of the exercises performed by the patient during rehabilitation at various stages during rehabilitation, including the start date of each exercise, its frequency, dosage, description, and rationale. All exercises were done while the tSCS applied at 30 Hz a range between 15-22 mA with each exercise sessions lasted between 30-40 minutes. By outlining the PT phases more clearly and specifying the progression of exercises, we aim to provide a more structured overview of the rehabilitation process and its integration with tSCS.

| **Exercise** | **Dosage** | **Rationale/Description** |
| --- | --- | --- |
| **1st Phase** | | |
| Suspended kickback  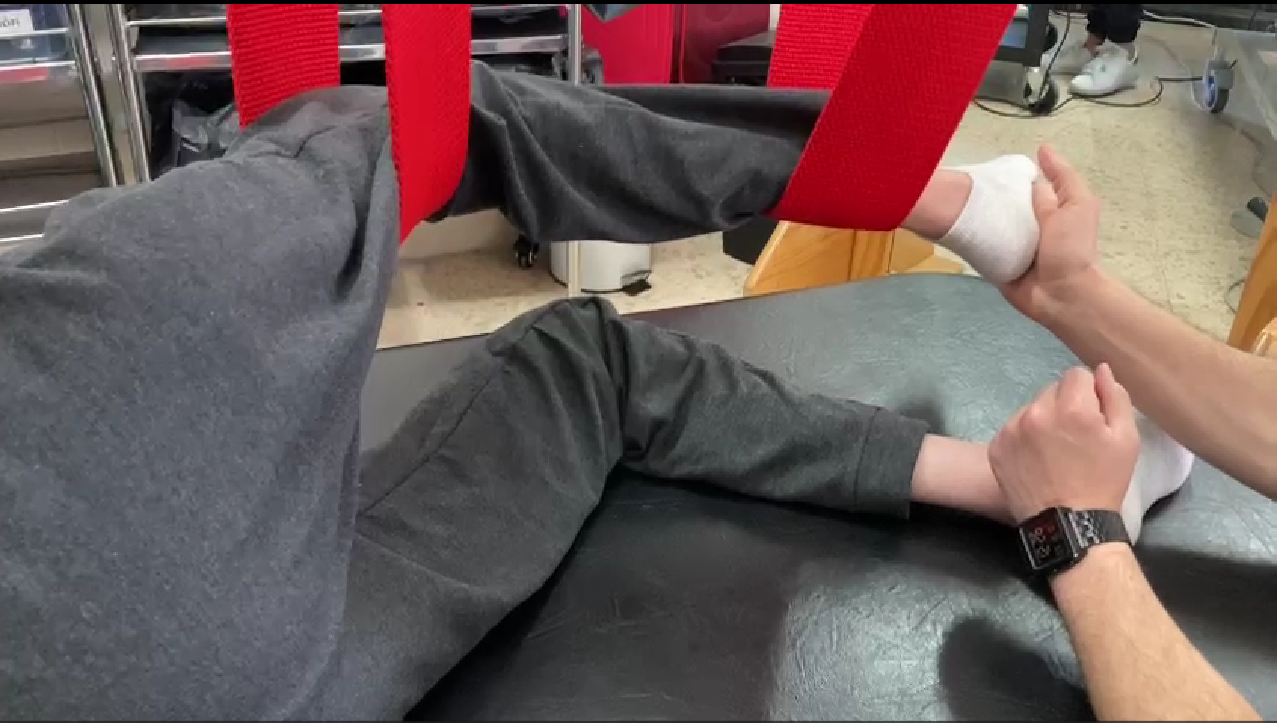 | 5 repetitions by 3 series per leg.  2 sessions per week | Motor output appeared after 8 months of tSCS alone. Consequently, a PT plan was implemented. In the initial stages of rehabilitation, we sought to train in triple extension, i.e. hip extension, knee extension, and plantar flexion. The exercise was performed sideways with the leg suspended to eliminate gravity and the patient was aided by a physiotherapist during the movement. |
| **2nd Phase** | | |
| Kneeling with Bosu ball  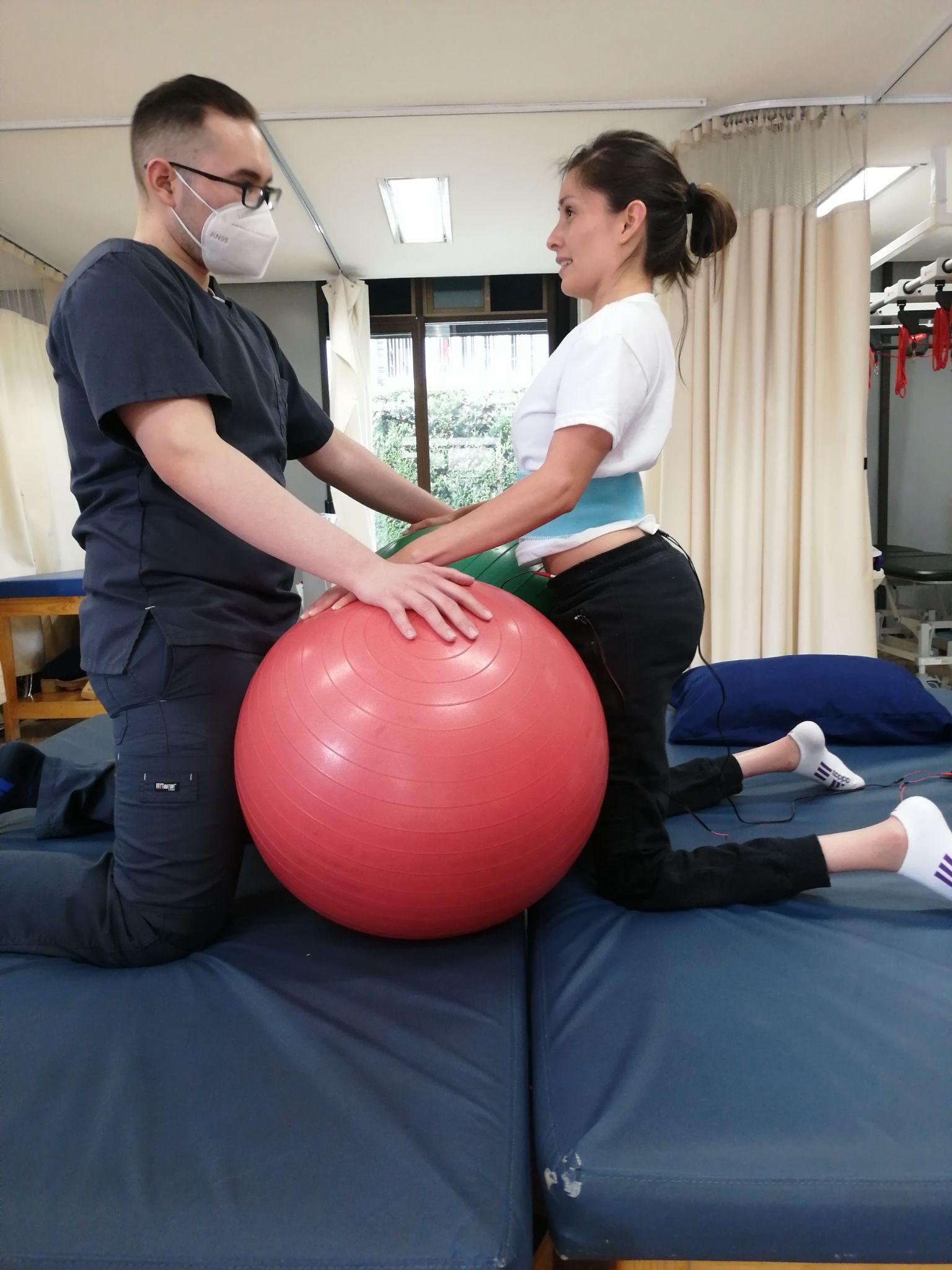 | By repetitions  5 repetitions by 3 series  By isometric contraction  20 seconds by 3 series  2 sessions per week | In this stage, we sought to explore verticalization in a partial way in the form of kneeling assisted with a Bosu ball. To do this, two exercises were performed, one done isometrically and the other by repetitions. In the isometric exercise, the patient was instructed to remain upright while raising each hand alternately. In the repetition exercise, the patient was instructed to kneel with the buttocks touching the heels and to stand up (as shown in the picture) leaning on the Bosu ball. |
| **3rd Phase** | | |
| ABD and ADD toe taps  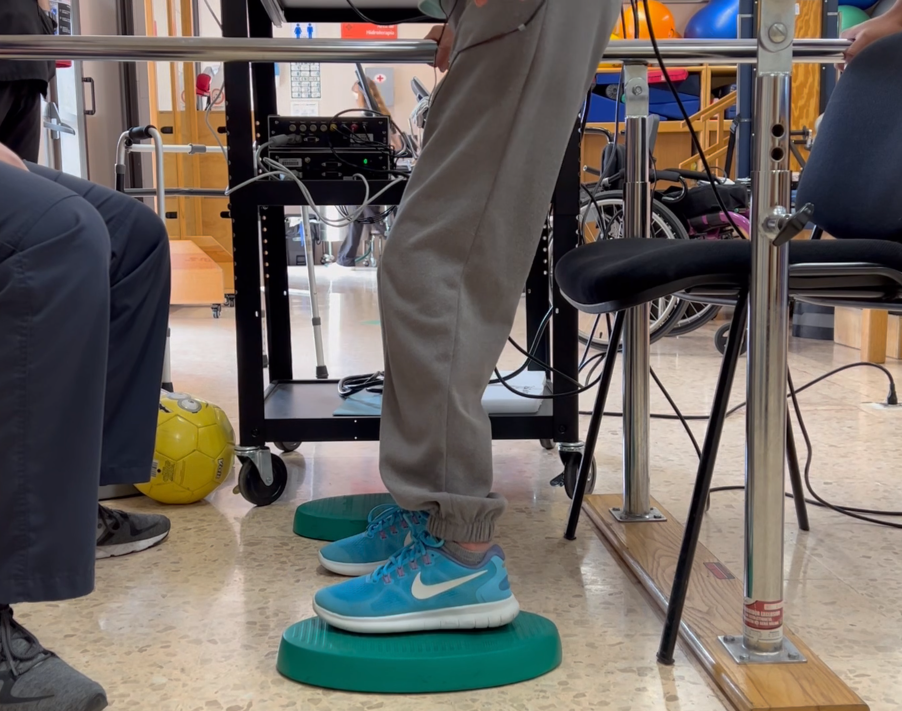 | 5 repetitions by 3 series per movement and leg  2 sessions per week | After achieving hip control and improved strength, it was decided to continue with a standing position with the help of parallel bars. Once the patient was in a standing position, the participant was asked to do hip abduction and adduction. |
| Ball kicks  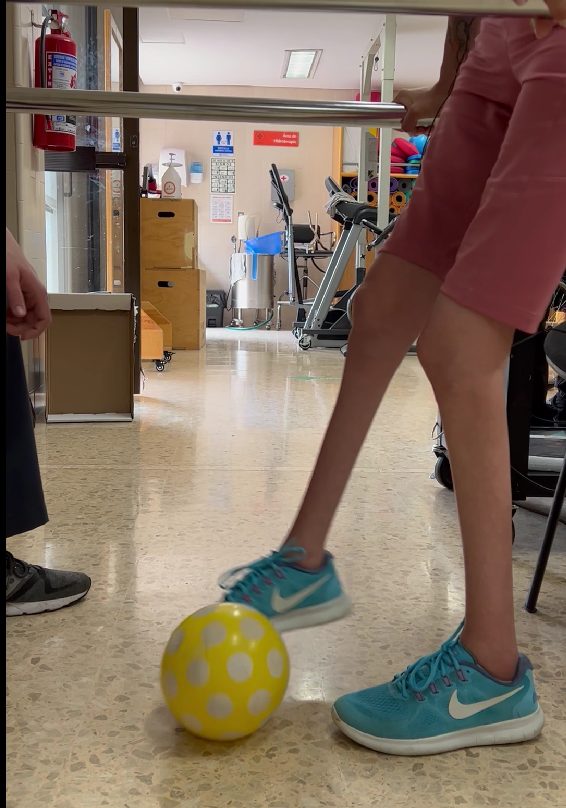 | 10 kicks by 3 series each leg  2 sessions per week | Exercises to improve coordination and hip flexion were implemented in this stage using the same setup with the parallel bars. The patient was asked to kick the ball as hard as possible. |
| **4th Phase** | | |
| Forward stepping  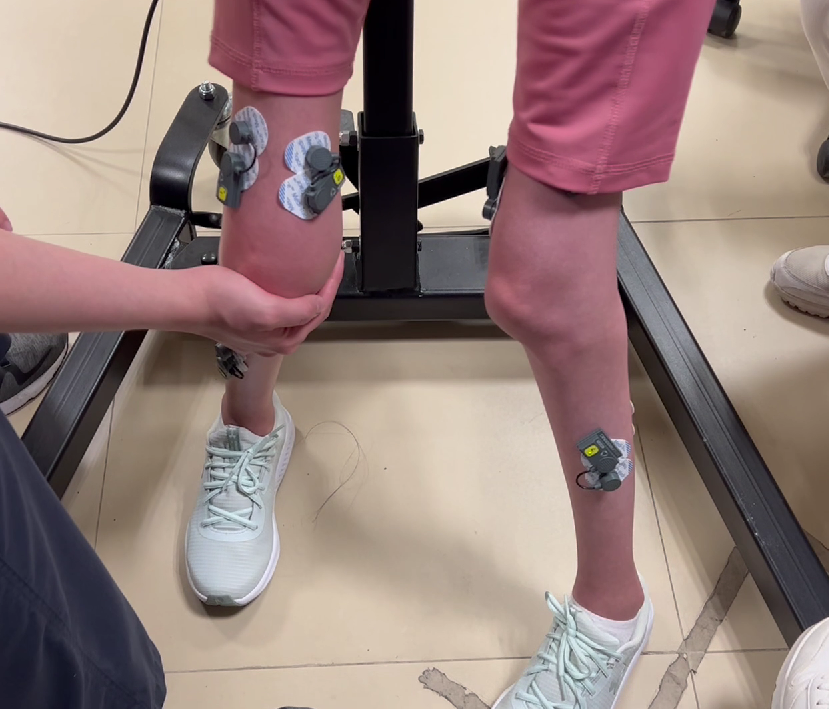 | 5 repetitions by 3 series each leg  2 sessions per week | After achieving hip flexion, abduction, and adduction, it was decided to take a step towards gait training. At this stage and thereafter, a body weight support machine was used to assist the patient in standing. During the exercises, the patient was asked to do hip flexion simulating a forward step while a physical therapist held the contralateral limb to provide stability. |
| Forward full step  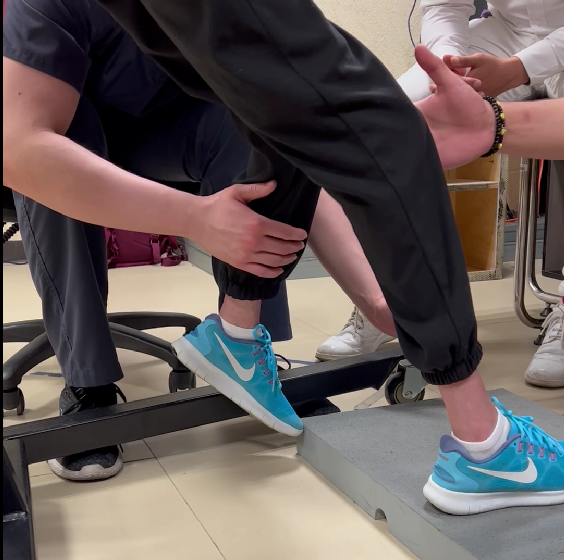 | 5 repetitions by 3 series for each leg  2 sessions per week | As we observed improvements in motor output in the hip muscles, we sought to take a step forward in the form of completing the full step during weight support and trying to involve quadriceps muscles. We used a small platform on which the participant put a foot on it while the other stayed behind. The exercise consisted of raising the back foot to the platform, simulating a full step. One physiotherapist supported the knee of the leg above the platform to avoid knee valgus while another physiotherapist assisted in the final stages of the movement of the leg behind as shown in the picture. |
| Bipedal pushdown  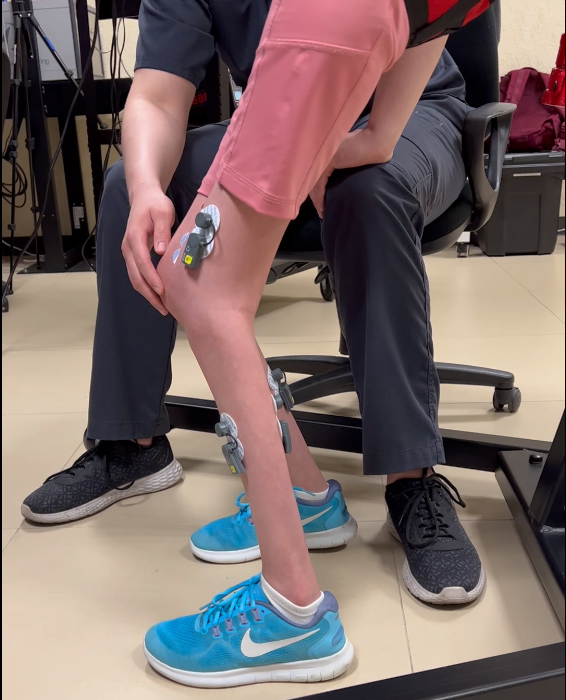 | 5 repetitions by 3 series  2 sessions per week | Using the same logic as the previous exercise, the patient was asked to push down with both feet during weight support. The participant was asked to think of the movement when getting up from a chair. |
| Bipedal side stepping  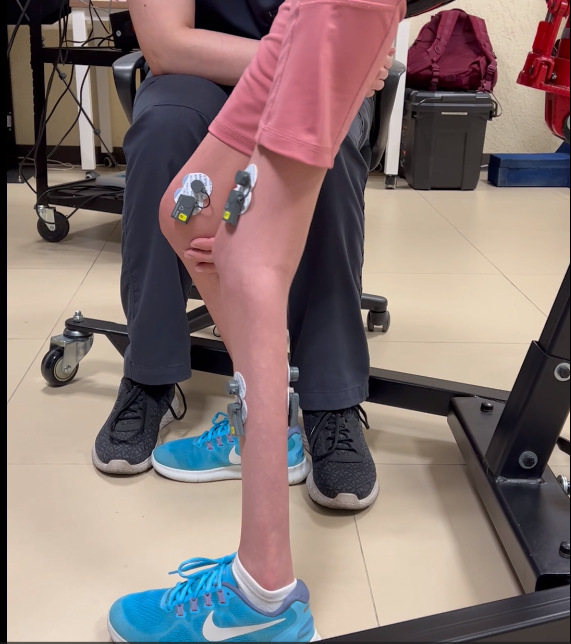 | 5 repetitions by 3 series each leg  2 sessions per week | To improve coordination and strength in hip muscles, the patient was asked to move the leg in and out in a controlled manner. A physical therapist supported the contralateral leg for knee stability. |

# ISNCSCI assessments from BL to 20-months follow-up. Sensory scores begin at T10 (Neurological level at baseline)

| **BASELINE** | | | | | | | | | |
| --- | --- | --- | --- | --- | --- | --- | --- | --- | --- |
| **RIGHT** | | | **SENSORY**  KEY SENSORY POINTS | | **LEFT** | | | **SENSORY**  KEY SENSORY POINTS | |
| **MOTOR**  KEY MUSCLES |  |  | LTR | PPR | **MOTOR**  KEY MUSCLES |  |  | LTL | PPL |
|  | T10 |  | 2 | 2 |  | T10 |  | 2 | 2 |
|  | T11 |  | 1 | 2 |  | T11 |  | 2 | 1 |
|  | T12 |  | 0 | 0 |  | T12 |  | 0 | 0 |
|  | L1 |  | 0 | 0 |  | L1 |  | 0 | 0 |
| *Hip flexors* | L2 | 0 | 0 | 0 | *Hip flexors* | L2 | 0 | 0 | 0 |
| *Knee extensors* | L3 | 0 | 0 | 0 | *Knee extensors* | L3 | 0 | 0 | 0 |
| *Ankle dorsiflexors* | L4 | 0 | 0 | 0 | *Ankle dorsiflexors* | L4 | 0 | 0 | 0 |
| *Long toe*  *extensors* | L5 | 0 | 0 | 0 | *Long toe*  *extensors* | L5 | 0 | 0 | 0 |
| *Ankle plantar*  *flexors* | S1 | 0 | 0 | 0 | *Ankle plantar flexors* | S1 | 0 | 0 | 0 |
| *Voluntary Anal Contraction (No)* | S2 | 0 | 0 | 0 | *Deep Anal Pressure*  *(No)* | S2 | 0 | 0 | 0 |
|  | S3 | 0 | 0 | 0 |  | S3 | 0 | 0 | 0 |
|  | S4-5 | 0 | 0 | 0 |  | S4-5 | 0 | 0 | 0 |

| **4 MONTHS** | | | | | | | | | |
| --- | --- | --- | --- | --- | --- | --- | --- | --- | --- |
| **RIGHT** | | | **SENSORY**  KEY SENSORY POINTS | | **LEFT** | | | **SENSORY**  KEY SENSORY POINTS | |
| **MOTOR**  KEY MUSCLES |  |  | LTR | PPR | **MOTOR**  KEY MUSCLES |  |  | LTL | PPL |
|  | T10 |  | 2 | 2 |  | T10 |  | 2 | 2 |
|  | T11 |  | 2 | 2 |  | T11 |  | 2 | 2 |
|  | T12 |  | 2 | 2 |  | T12 |  | 2 | 2 |
|  | L1 |  | 1 | 0 |  | L1 |  | 0 | 0 |
| *Hip flexors* | L2 | 0 | 1 | 1 | *Hip flexors* | L2 | 0 | 1 | 1 |
| *Knee extensors* | L3 | 0 | 1 | 0 | *Knee extensors* | L3 | 0 | 1 | 1 |
| *Ankle dorsiflexors* | L4 | 0 | 1 | 0 | *Ankle dorsiflexors* | L4 | 0 | 1 | 1 |
| *Long toe*  *extensors* | L5 | 0 | 0 | 1 | *Long toe*  *extensors* | L5 | 0 | 1 | 1 |
| *Ankle plantar*  *flexors* | S1 | 0 | 0 | 1 | *Ankle plantar flexors* | S1 | 0 | 0 | 1 |
| *Voluntary Anal Contraction (No)* | S2 | 0 | 0 | 0 | *Deep Anal Pressure*  *(No)* | S2 | 0 | 1 | 0 |
|  | S3 | 0 | 0 | 0 |  | S3 | 0 | 0 | 1 |
|  | S4-5 | 0 | 0 | 0 |  | S4-5 | 0 | 0 | 0 |

| **8 MONTHS** | | | | | | | | | |
| --- | --- | --- | --- | --- | --- | --- | --- | --- | --- |
| **RIGHT** | | | **SENSORY**  KEY SENSORY POINTS | | **LEFT** | | | **SENSORY**  KEY SENSORY POINTS | |
| **MOTOR**  KEY MUSCLES |  |  | LTR | PPR | **MOTOR**  KEY MUSCLES |  |  | LTL | PPL |
|  | T10 |  | 2 | 2 |  | T10 |  | 2 | 2 |
|  | T11 |  | 2 | 2 |  | T11 |  | 2 | 2 |
|  | T12 |  | 2 | 2 |  | T12 |  | 2 | 2 |
|  | L1 |  | 1 | 1 |  | L1 |  | 2 | 2 |
| *Hip flexors* | L2 | 1 | 0 | 0 | *Hip flexors* | L2 | 1 | 1 | 1 |
| *Knee extensors* | L3 | 0 | 0 | 0 | *Knee extensors* | L3 | 0 | 0 | 0 |
| *Ankle dorsiflexors* | L4 | 0 | 0 | 0 | *Ankle dorsiflexors* | L4 | 0 | 0 | 0 |
| *Long toe*  *extensors* | L5 | 0 | 0 | 0 | *Long toe*  *extensors* | L5 | 0 | 0 | 0 |
| *Ankle plantar*  *flexors* | S1 | 0 | 0 | 0 | *Ankle plantar flexors* | S1 | 0 | 0 | 0 |
| *Voluntary Anal Contraction (No)* | S2 | 0 | 0 | 0 | *Deep Anal Pressure*  *(No)* | S2 | 0 | 0 | 0 |
|  | S3 | 0 | 0 | 0 |  | S3 | 0 | 0 | 0 |
|  | S4-5 | 0 | 0 | 0 |  | S4-5 | 0 | 0 | 0 |

| **12 MONTHS** | | | | | | | | | |
| --- | --- | --- | --- | --- | --- | --- | --- | --- | --- |
| **RIGHT** | | | **SENSORY**  KEY SENSORY POINTS | | **LEFT** | | | **SENSORY**  KEY SENSORY POINTS | |
| **MOTOR**  KEY MUSCLES |  |  | LTR | PPR | **MOTOR**  KEY MUSCLES |  |  | LTL | PPL |
|  | T10 |  | 2 | 2 |  | T10 |  | 2 | 2 |
|  | T11 |  | 2 | 2 |  | T11 |  | 2 | 2 |
|  | T12 |  | 2 | 2 |  | T12 |  | 2 | 2 |
|  | L1 |  | 1 | 1 |  | L1 |  | 2 | 2 |
| *Hip flexors* | L2 | 3 | 1 | 1 | *Hip flexors* | L2 | 2 | 1 | 1 |
| *Knee extensors* | L3 | 0 | 1 | 1 | *Knee extensors* | L3 | 0 | 1 | 0 |
| *Ankle dorsiflexors* | L4 | 0 | 0 | 0 | *Ankle dorsiflexors* | L4 | 0 | 0 | 0 |
| *Long toe*  *extensors* | L5 | 0 | 0 | 0 | *Long toe*  *extensors* | L5 | 0 | 0 | 0 |
| *Ankle plantar*  *flexors* | S1 | 0 | 0 | 0 | *Ankle plantar flexors* | S1 | 0 | 0 | 0 |
| *Voluntary Anal Contraction (No)* | S2 | 0 | 0 | 0 | *Deep Anal Pressure*  *(No)* | S2 | 0 | 0 | 0 |
|  | S3 | 0 | 0 | 0 |  | S3 | 0 | 0 | 0 |
|  | S4-5 | 0 | 0 | 0 |  | S4-5 | 0 | 0 | 0 |

| **16 MONTHS** | | | | | | | | | |
| --- | --- | --- | --- | --- | --- | --- | --- | --- | --- |
| **RIGHT** | | | **SENSORY**  KEY SENSORY POINTS | | **LEFT** | | | **SENSORY**  KEY SENSORY POINTS | |
| **MOTOR**  KEY MUSCLES |  |  | LTR | PPR | **MOTOR**  KEY MUSCLES |  |  | LTL | PPL |
|  | T10 |  | 2 | 2 |  | T10 |  | 2 | 2 |
|  | T11 |  | 2 | 2 |  | T11 |  | 2 | 2 |
|  | T12 |  | 2 | 2 |  | T12 |  | 2 | 2 |
|  | L1 |  | 2 | 1 |  | L1 |  | 1 | 1 |
| *Hip flexors* | L2 | 3 | 1 | 1 | *Hip flexors* | L2 | 2 | 1 | 1 |
| *Knee extensors* | L3 | 0 | 1 | 1 | *Knee extensors* | L3 | 0 | 1 | 1 |
| *Ankle dorsiflexors* | L4 | 0 | 1 | 0 | *Ankle dorsiflexors* | L4 | 0 | 1 | 0 |
| *Long toe*  *extensors* | L5 | 0 | 1 | 1 | *Long toe*  *extensors* | L5 | 0 | 0 | 1 |
| *Ankle plantar*  *flexors* | S1 | 0 | 1 | 1 | *Ankle plantar flexors* | S1 | 0 | 0 | 0 |
| *Voluntary Anal Contraction (No)* | S2 | 0 | 0 | 1 | *Deep Anal Pressure*  *(No)* | S2 | 0 | 0 | 1 |
|  | S3 | 0 | 0 | 0 |  | S3 | 0 | 0 | 0 |
|  | S4-5 | 0 | 0 | 0 |  | S4-5 | 0 | 0 | 0 |

| **20 MONTHS** | | | | | | | | | |
| --- | --- | --- | --- | --- | --- | --- | --- | --- | --- |
| **RIGHT** | | | **SENSORY**  KEY SENSORY POINTS | | **LEFT** | | | **SENSORY**  KEY SENSORY POINTS | |
| **MOTOR**  KEY MUSCLES |  |  | LTR | PPR | **MOTOR**  KEY MUSCLES |  |  | LTL | PPL |
|  | T10 |  | 2 | 2 |  | T10 |  | 2 | 2 |
|  | T11 |  | 2 | 2 |  | T11 |  | 2 | 2 |
|  | T12 |  | 2 | 2 |  | T12 |  | 2 | 2 |
|  | L1 |  | 1 | 1 |  | L1 |  | 1 | 1 |
| *Hip flexors* | L2 | 3 | 1 | 1 | *Hip flexors* | L2 | 2 | 1 | 1 |
| *Knee extensors* | L3 | 1 | 1 | 1 | *Knee extensors* | L3 | 1 | 1 | 1 |
| *Ankle dorsiflexors* | L4 | 0 | 0 | 1 | *Ankle dorsiflexors* | L4 | 0 | 0 | 1 |
| *Long toe*  *extensors* | L5 | 0 | 0 | 1 | *Long toe*  *extensors* | L5 | 0 | 0 | 1 |
| *Ankle plantar*  *flexors* | S1 | 0 | 0 | 1 | *Ankle plantar flexors* | S1 | 0 | 0 | 1 |
| *Voluntary Anal Contraction (No)* | S2 | 0 | 0 | 1 | *Deep Anal Pressure*  *(No)* | S2 | 0 | 0 | 1 |
|  | S3 | 0 | 0 | 1 |  | S3 | 0 | 0 | 1 |
|  | S4-5 | 0 | 0 | 0 |  | S4-5 | 0 | 0 | 0 |

# EMG electrodes placement

Placement of the electrodes for EMG recording, corresponding to Figures 2 and 3. Muscles were recorded bilaterally in gluteus medialis (red), tensor fascia latae (blue), rectus femoris (green), sartorius (yellow), biceps femoris (black), tibialis anterior (orange) and medial gastrocnemius (purple). Vastus medialis (not shown) was also recorded but showed no activity.


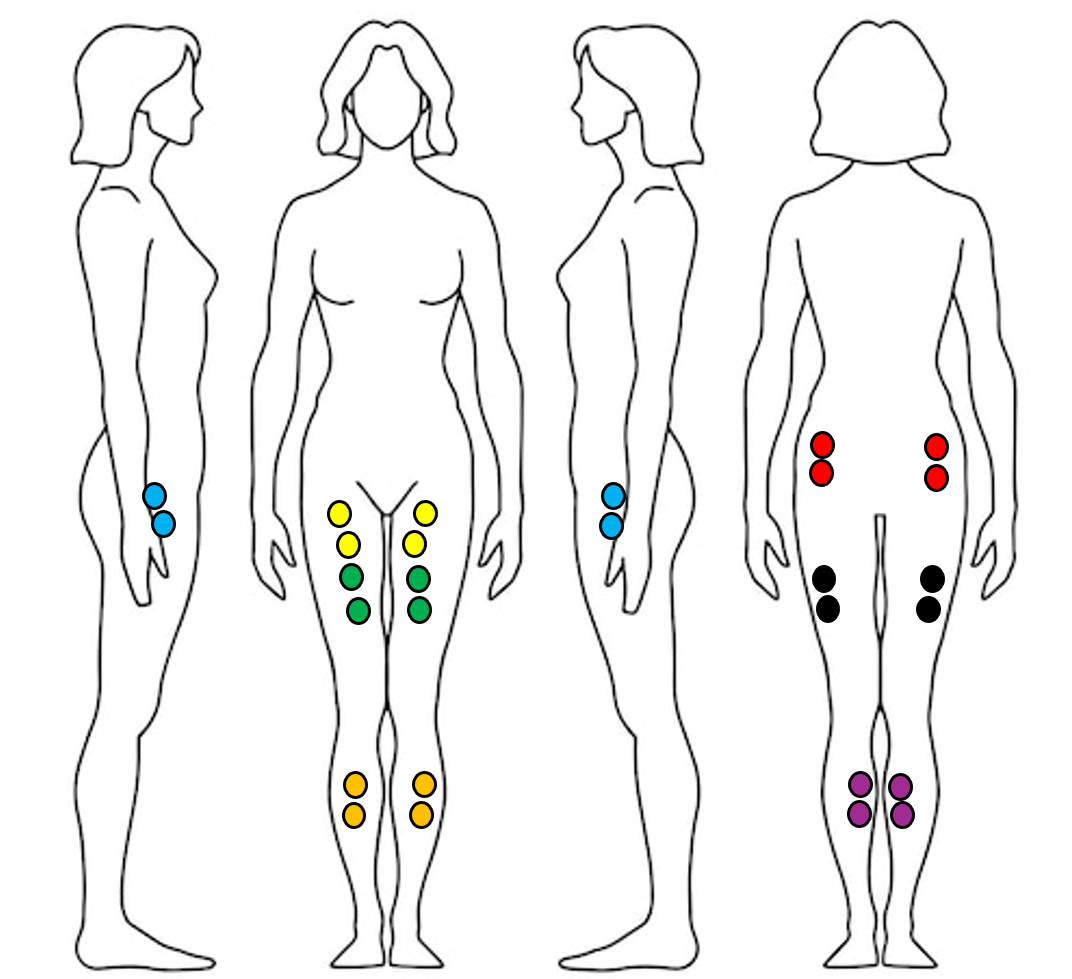


1. **Stimulus Artifact removal process.**

Spectral analysis of EMG recordings during “tSCS off” and “tSCS on”, corresponding to overground stepping as shown in Figure 3A-B. Left RF is shown as example. **A.** “tSCS off”. Post-processing eliminated a 60 Hz noise, confirmed by the spectral analysis in the plot on the right. B. “tSCS on”. Stimulus artifact at 30 Hz and its harmonics are shown in the spectral analysis. After post-processing and artifact removal, harmonics are eliminated, and EMG components emerge. A persistent 30 Hz component is visible, but its amplitude was attenuated. Note that the corresponding Fourier spectrum scale was adjusted to visualize EMG components.


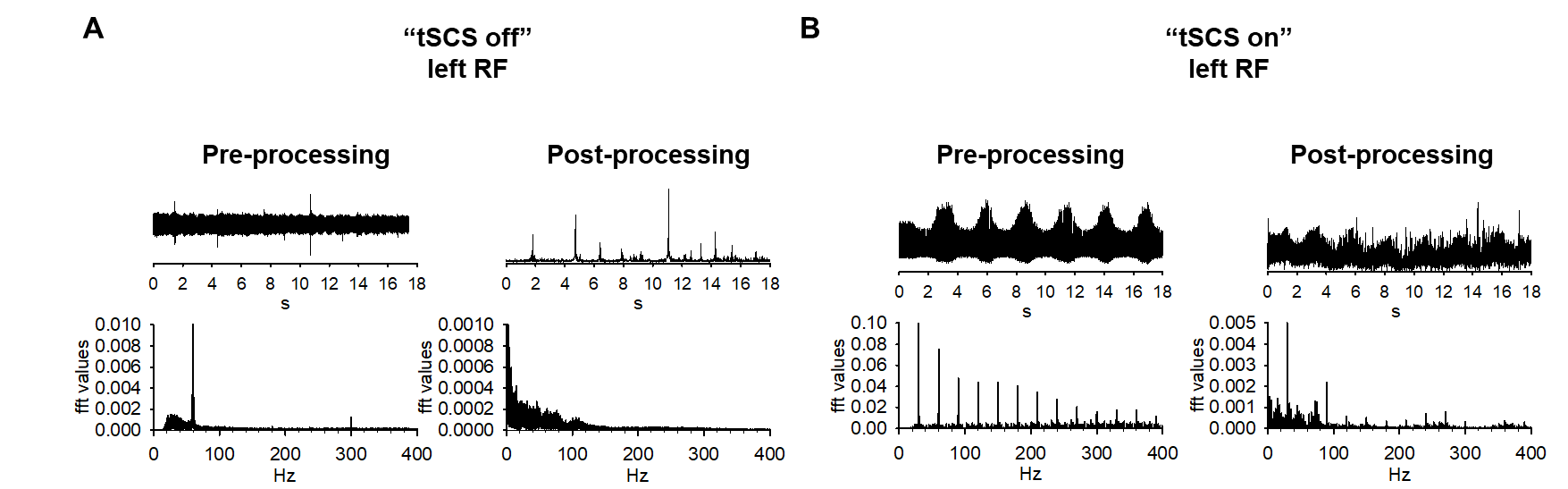

Supplement: Supplementary file 1 [file Data_Sheet_1.docx]
